# The PLCE1 rs2274223 variant is associated with the risk of laryngeal squamous cell carcinoma

**DOI:** 10.7150/ijms.49012

**Published:** 2020-10-08

**Authors:** Yi Zhang, Wei Li, Ying Wang, Ningyu Wang

**Affiliations:** 1 Department of Otolaryngology-Head and Neck Surgery, Beijing Chaoyang Hospital, Capital Medical University, Beijing, China.; 2 The Department of Head and Neck surgery, Hubei Cancer Hospital, Wuhan, Hubei, China.; 3 Department of Pathology, Beijing Chaoyang Hospital, Capital Medical University, Beijing, China.

**Keywords:** Laryngeal squamous cell carcinoma (LSCC), single nucleotide polymorphism (SNP), PLCE1, rs2274223

## Abstract

**Background:** Laryngeal squamous cell carcinoma (LSCC) ranks second in the mortality rate in respiratory malignant tumors and has potential similarity in genomic alterations with the esophageal squamous cell carcinoma (ESCC). The PLCE1 rs2274223 variant is the most significant susceptibility loci identified in ESCC. Whether it is also associated with LSCC susceptibility is still unclear.

**Materials and Methods:** A total of 331 LSCC patients and 349 healthy controls were recruited in this study. The PLCE1 rs2274223 variant was genotyped by using the Taqman SNP Genotyping Assay. Association between PLCE1 rs2274223 variant and LSCC risk was estimated by logistic regression analysis, which was performed using SAS software.

**Results:** The PLCE1 rs2274223 variant was identified to be significantly associated with the susceptibility of LSCC in the additive model (OR = 1.40, 95% CI: 1.06-1.86, *P*=0.019). Compared with the wild-type (AA) carriers, the risk genotype (GG) carriers had a 2.8-fold risk of LSCC (95% CI: 1.13-7.06, *P*=0.026). Stratified analysis showed that the association between rs2274223 and LSCC risk was with higher significance in individuals above 60 (P = 0.027) males (P = 0.030) or non-smokers (P = 0.026).

**Conclusion:** The PLCE1 rs2274223 variant was significantly associated with risk of LSCC, which may be a potential biomarker and therapeutic target for the LSCC.

## Introduction

Laryngeal cancer is one of most common head and neck cancers, the majority of cases are laryngeal squamous cell carcinoma (LSCC) 1. This tumor type ranks second in the mortality rate in respiratory malignant tumors 2, 3. Genetic and environmental factors contribute to the disease, and genetic variation is one of the most promoting factors that accounting for its pathogenesis 4, 5. Another squamous cell carcinoma (SCC) in the upper aerodigestive tract, the esophageal squamous cell carcinoma (ESCC), has many similarities in the genomic alteration and susceptibility genes with LSCC.

Phospholipase C epsilon 1 gene (PLCE1) is a C isoenzyme of phosphorus esterase, which was first identified in C. elegant by Shibatohge M and colleges 6, and its gene is located in chromosome 10q23 in human 7. It plays an important role in cell growth, differentiation and oncogenesis 8. Different expression profiles of PLCE1, like overexpression or down-regulation, could be found in different types of cancer, and functioned in diverse manners 9-12. Large scale genome-wide association (GWAS) studies from different groups had demonstrated that the rs2274223, located in exon 26 of PLCE1, was strongly associated with the risk of ESCC 13-17. The PLCE1 polymorphisms and its distribution have been reported to be involved in its gene expression and tumor development 18. The PLCE1 rs2274223 is of particular interest and attention for its association with susceptibility of many types of cancer, like colorectal cancer 19, gastric cancer 18, 19, esophageal cancer, and several other digestive tract cancers 8, 20-24. Although the PLCE1 rs2274223 has been reported to affect many types of digestive tract cancers, whether the PLCE1 rs2274223 A/G variant is associated with LSCC susceptibility is still unclear.

The above summary suggested that role of PLCE1 and rs2274223 had been extensively studied in diverse cancer types, especially in ESCC. In the present study, we genotyped the PLCE1 rs2274223 A/G polymorphism with 331 LSCC patients and 349 healthy controls and analyzed the association between the PLCE1 rs2274223 and LSCC susceptibility. Our work will give novel insights into the PLCE1 rs2274223 variant as a biomarker for the early detection of LSCC.

## Materials and Methods

### Study subjects

In this study, we enrolled 331 LSCC patients and 349 healthy controls from Hubei Cancer Hospital and Beijing Chaoyang Hospital between March 2013 to December 2018. The LSCC patients were diagnosed histopathologically by at least two pathologists according to the World Health Organization classification. The controls were cancer-free individuals who seeking routine physical examination in the same hospital as patients were recruited. All participants had written informed consent at recruitment. There is about 45% power to identify significant results with estimated odds ratio being 1.40 and the minor allele frequency of variant in controls being 0.20. The demographic characteristics like sex, age, smoking and drinking status were obtained from the medical records. The study was approved by Hubei Cancer Hospital Ethics Committee with KYLLB2017002.

### SNP genotyping

Blood samples from LSCC patients and healthy controls were collected and stored at -80 Celsius degree, and the whole genome DNA were extracted for genotyping analysis by using TIANamp Genomic DNA Kit (Tiangen Biotech, China). The PLCE1 rs2274223 picked up from ESCC GWAS analysis was selected for genotyping, and the variant was genotyped by using the Taqman SNP Genotyping Assay (Applied Biosystems, USA).

### Statistical analysis

Logistic regression analysis adjusted for sex, age, smoking and drinking status were used to assess the strength of association between the PLCE1 rs2274223 and LSCC risk. All statistical analyses were performed using SAS software (version 9.1; SAS Institute, Cary, NC) and SNPStats app 25. A *P* value <0.05 was considered as statistically significant and all tests were two-sided.

## Results

### Study sample processing and the characteristics of subjects

The basic demographic, smoking and drinking status of LSCC patients and healthy controls are listed in the **Table 1**. The current study recruited 331 LSCC patients and 349 healthy controls, with mean age of 60.42 and 62.02, respectively. There are 308 males and 23 females in LSCC group, 295 males and 54 females in healthy control group. Smoking and drinking factors are thought to be involved in gene mutation and cancer development. Therefore, we also collected and listed the related information of subjects in our study (**Table 1**).

### The PLCE1 rs2274223 genotypes associates with the susceptibility of LSCC

In our study, the PLCE1 rs2274223 was selected for genotyping. The number and the percentage of LSCC patients with AA, AG and GG genotypes were 178 (53.8%), 135 (40.8%) and 18 (5.4%), respectively (**Table 2**). In healthy control group, these data were AA, 216 (61.9%), AG 125 (35.8%), and GG, 8 (2.3%), respectively (**Table 2**). The Hardy-Weinberg balance analysis showed that the allelic distributions in cases and controls were in equilibrium (*P* > 0.01). The odds ratio (OR) and corresponding 95% confidence intervals (CIs) adjusted for sex, age, smoking and drinking status for each genotype was used to assess the associations between PLCE1 rs2274223 and LSCC risk by using unconditional multivariate logistic regression analysis. The OR, 95% CIs, and the corresponding *p* value to each genotype were listed in **Table 3.** The results showed that when comparing to AA genotype carriers, the GG genotype carriers were significantly associated with higher LSCC risk with OR being 2.82 (95% CI = 1.13-7.06, *P* = 0.026). Besides, by using the additive model, the results demonstrated that the rs2274223 G allele carriers was significantly associated with higher LSCC risk comparing to the rs2274223 A allele carriers with OR being 1.40 (95% CI = 1.06-1.86, *P* = 0.019) (**Table 3**).

### Stratified analysis showed the association between PLCE1 rs2274223 and LSCC risk was with higher significance in individuals elder 60, males or non-smokers

The above data indicated that the PLCE1 rs2274223 variant was significantly associated with LSCC risk. As subject characteristics like age, sex, smoking and drinking status are all cancer-related factors 26-28, we next tested the associations by stratified each of the above factors (**Table 3**). The results suggested that the association between the PLCE1 rs2274223 and LSCC risk was with higher significance in individuals elder than 60 (OR =1.50, 95% CI = 1.05-2.15, *P*=0.027), males (OR =1.39, 95% CI = 1.03-1.86, *P* = 0.030) or nonsmokers (OR = 2.00, 95% CI = 1.09-3.69, *P* = 0.026). The association between the PLCE1 rs2274223 and LSCC risk were not significance in individuals less than 60 years old, females or smokers. The association between the PLCE1 rs2274223 and LSCC risk were not significance in both drinkers and nondrinkers, which may due to relatively small sample size in this study. No significant interaction was observed for rs2274223 and sex, age, smoking status or drinking status (**Table 4**).

## Discussion

Investigation of tumor susceptibility is of particular significance for cancer evaluation and therapy 29, and many SNPs had been extensively confirmed to be associated with tumor susceptibility 30-34. Our current study focused on the association between LSCC risk and PLCE1 rs2274223, a very hot and promising biomarker for upper digestive cancers, especially ESCC.

After collecting 331 LSCC patients and 349 healthy controls samples and genotyped with PLCE1 rs2274223, we found that the PLCE1 rs2274223-G was significantly associated with a higher LSCC risk, which is similar with its association in ESCC 13-17. Further stratified analysis demonstrated that age, sex, and smoking status may affect the association. However, the PLCE rs2274223variant exhibited different behaviors in the susceptibility of different cancer types. For example, the PLCE1 rs2274223 A/G might reduce gene expression and the G allele might contribute to a higher risk of colorectal cancer 19. And the bladder cancer development was suggested to be related to increased PLCE1 expression 9, and overexpression of PLCE1 also positively affected on transfer of the head and neck squamous cell carcinoma 10. Therefore, whether and how the PLCE1 rs2274223 altered the PLCE1 expression paten in LSCC patients and the LSCC development need to be investigated in the future.

There were still several limitations in this study at current stage. Firstly, the sample size of this study was relatively small with limited power. More subjects should be recruited in the future study to replicate the association. Besides, subjects from different region may exhibit different sensitivity, so large multicenter study should be performed, and more comprehensive analysis should be carried out. Finally, cancer development was always driven by multiple genes mutations, so analysis of PLCE1 rs2274223 combining with other SNP on affecting LSCC susceptibility should be performed in following study.

In summary, this study identified that the rs2274223 variant in PLCE1 was significantly associated with the susceptibility of LSCC. This variant may serve as a potential biomarker for the early detection of LSCC.

## Figures and Tables

**Table 1:** Characteristics summary of study subjects

	LSCC (n=331)	Controls (n=349)	*P* values*
Age (years), Mean ±SD	60.42±10.00	62.02±8.61	
**Gender, n (%)**			0.0004
Male	308 (93.1)	295 (84.5)	
Female	23 (6.9)	54 (15.5)	
**Smoking status**			< 0.0001
Nonsmoker, n (%)	46 (13.9)	145 (41.5)	
Smoker, n (%)	285 (86.1)	204 (58.5)	
**Drinking status**			< 0.0001
Nondrinker, n (%)	111 (33.5)	193 (55.3)	
Drinker, n (%)	220 (66.5)	156 (44.7)	

*P* values are calculated by using chi-squared test.

**Table 2:** Allele frequencies and exact test for Hardy-Weinberg equilibrium of rs2274223

	A/A Count (Proportion)	A/G Count (Proportion)	G/G Count (Proportion)	A Count (Proportion)	G Count (Proportion)	*P* value
All subjects	394 (0.58)	260 (0.38)	26 (0.04)	1048 (0.77)	312 (0.23)	0.039
Controls	216 (0.62)	125 (0.36)	8 (0.02)	557 (0.80)	141 (0.20)	0.045
Cases	178 (0.54)	135 (0.41)	18 (0.05)	491 (0.74)	171 (0.26)	0.310

*P* values are calculated by using exact test for Hardy-Weinberg equilibrium.

**Table 3:** Summary of PLCE1 rs2274223 and its association with risk of LSCC

Model	Genotype	Controls	Cases	OR (95% CI)	*P* value
	A/A	216 (61.9%)	178 (53.8%)	1.00	
	A/G	125 (35.8%)	135 (40.8%)	1.28 (0.92-1.79)	0.146
	G/G	8 (2.3%)	18 (5.4%)	2.82 (1.13-7.06)	0.026
Dominant	A/A	216 (61.9%)	178 (53.8%)	1.00	0.056
	A/G-G/G	133 (38.1%)	153 (46.2%)	1.37 (0.99-1.90)	
Recessive	A/A-A/G	341 (97.7%)	313 (94.6%)	1.00	0.035
	G/G	8 (2.3%)	18 (5.4%)	2.56 (1.03-6.34)	
Overdominant	A/A-G/G	224 (64.2%)	196 (59.2%)	1.00	0.260
	A/G	125 (35.8%)	135 (40.8%)	1.21 (0.87-1.68)	
Log-additive	---	---	---	1.40 (1.06-1.86)	0.019

OR, odds ratio; CI, confidence interval. *P* values are two sided and were calculated in logistic regression analysis adjusted for sex, age, smoking status and drinking status. Significant results were in bold.

**Table 4:** Summary of stratified analysis of associations between rs2274223 and LSCC risk in additive model

	MAF Cases	MAF Controls	OR (95% CI)	*P* value*	Interaction* P* value
**Age, years**					
> 60	**0.271**	**0.197**	**1.50 (1.05-2.15)**	**0.027**	0.677
≤ 60	0.245	0.214	1.23 (0.75-2.00)	0.408	
**Gender**					
Male	**0.255**	**0.197**	**1.39 (1.03-1.86)**	**0.030**	0.798
Female	0.304	0.231	1.80 (0.56-5.80)	0.323	
**Smoking status**					
Smoker	0.254	0.208	1.31 (0.94-1.82)	0.110	0.483
Nonsmoker	**0.283**	**0.193**	**2.00 (1.09-3.69)**	**0.026**	
**Drinking status**					
Drinker	0.268	0.208	1.45 (0.99-2.14)	0.057	0.945
Nondrinker	0.239	0.197	1.38 (0.89-2.15)	0.153	

MAF, minor allele frequency; OR, odds ratio; CI, confidence interval. *P values are two sided and were calculated in logistic regression analysis adjusted for sex, age, smoking status and drinking status. The interaction* P* values were calculated by conducting a one degree-of-freedom Wald test of a single interaction parameter as implemented in an unconditional logistic regression with age, sex, smoking status or drinking status as covariates.

## Abbreviations

PLCE1: phospholipase C epsilon 1
LSCC: laryngeal squamous cell carcinoma
OR: odds ratio
95% CI: 95% confidence interval
GWAS: genome-wide association study
SNP: single nucleotide polymorphism
